# Hyperhomocysteinemia in men with a reproductive history of fetal neural tube defects: Three case reports and literature review

**DOI:** 10.1097/MD.0000000000013998

**Published:** 2019-01-11

**Authors:** Yang Yu, Chunshu Jia, Qingyang Shi, Yueying Zhu, Yanhong Liu

**Affiliations:** Center for Reproductive Medicine and prenatal diagnosis, First Bethune Hospital of Jilin University, Changchun, China.

**Keywords:** hyperhomocysteinemia, men, methylenetetrahydrofolate reductase gene, neural tube defects

## Abstract

**Rationale:**

Hereditary hyperhomocysteinemia results from a polymorphism in the methylenetetrahydrofolate reductase (MTHFR) gene that reduces folate metabolism. Mutations in the MTHFR gene are common in parents who have given birth to children with neural tube defects (NTDs). Most research has focused on the risk for fetal NTDs in women with hyperhomocysteinemia and MTHFR gene mutations. Studies investigating the association between hyperhomocysteinemia, MTHFR gene mutations, and the risk for fetal NTDs in men are scarce.

**Patient concerns:**

Here, we report on 3 men with hyperhomocysteinemia and the MTHFR C677T homozygous TT genotype that have reproductive histories of fetal NTDs.

**Diagnosis:**

these 3 men were diagnosed as hyperhomocysteinemia and MTHFR C677T homozygous TT genotype.

**Interventions:**

Three men received homocysteine-lowering therapy.

**Outcomes:**

The first man's wife became pregnant, and a healthy infant was spontaneously delivered at term, the other 2 men's wives are still not pregnant.

**Lessons:**

Findings from this case reports and published literature imply that hereditary hyperhomocysteinemia in men affects sperm quality and sperm DNA methylation and causes epigenetic modifications that can result in fetal NTDs. We recommend monitoring homocysteine and folate levels in men before conception and supplementing with folate as needed, especially in men with a reproductive history of fetuses with neural tube or other birth defects.

## 1 Introduction

Fetal neural tube defects (NTDs) are congenital defects that are caused by the incomplete closure of the neural tube during early embryonic development. The 3 most common types of NTDs are anencephalus, spina bifida, and meningocele.^[1]^

Current evidence indicates an association between hyperhomocysteinemia in women and fetal NTDs. Finkelstein^[2]^ reported that hyperhomocysteinemia may potentially affect embryonic development by interfering with the embryonic cell cycle or inducing apoptosis. Rosenquist et al^[3]^ showed that hyperhomocysteinemia increases the number of NTDs in chick embryos. Brouns et al^[4]^ demonstrated that homocysteine exposure can delay neural tube closure in avian embryos and speculated that hyperhomocysteinemia exerts a similar effect in the human fetus. Taparia et al^[5]^ suggested that homocysteinemia indirectly influences NTD development. Boxmeer^[6]^ considered that hyperhomocysteinemia has direct cytotoxicity and leads to overproduction of reactive oxygen species, inhibition of transmethylation, and abnormal gene expression. Van der Put et al hypothesized that hyperhomocysteinemia resulting from methylenetetrahydrofolate reductase (MTHFR) deficiency causes adverse reproductive outcomes such as fetal NTDs.^[7]^

A large-scale, prospective, randomized, double-blind study in 33 centers in 7 countries in 1991 by the Medical Research Council Vitamin Group demonstrated that folate supplementation in women can reduce the recurrence risk of fetal NTDs in mothers with a previously affected pregnancy.^[8]^ Organizations such as the American Congress of Obstetricians and Gynecologists (ACOG) and Society of Obstetricians and Gynaecologists of Canada (SOGC) recommend supplementary use of oral folate in women throughout pregnancy to reduce the incidence of fetal NTDs.^[9,10]^ Internationally there is consensus that folate supplementation in women during pregnancy can reduce the incidence of fetal NTDs; however, there are no recommendations for preconception oral folate supplementation in men.

Hyperhomocysteinemia may be hereditary or acquired. Hereditary hyperhomocysteinemia results from a mutation in the MTHFR gene that reduces folate metabolism. Mutations in the MTHFR gene are common in parents who have given birth to children with NTDs, and their offspring. For example, in the Netherlands, 14% to 16% of mothers and 10% to 15% of fathers of children with spina bifida, and 3% to 18% of those children had a homozygous mutation in the MTHFR gene, while the incidence of this mutation in the normal population was only 5%.^[11–13]^ Acquired hyperhomocysteinemia is caused by insufficient intake of dietary folate, Vitamin B6 or Vitamin B12, and/or the use of certain medications.

Published literature is replete with reports on the associated risk for fetal NTDs in women with hyperhomocysteinemia and MTHFR gene mutations. However, studies investigating the association between hyperhomocysteinemia, MTHFR gene mutations, and the risk for fetal NTDs in men are scarce. Here, we report on 3 men with hyperhomocysteinemia and reproductive histories of fetal NTDs.

## 2 Case reports

The study protocol was approved by the Ethics Committee of the First Hospital of Jilin University and written informed consent was obtained from all patients.

### 2.1 Case 1

A 28-year-old man and a former wife underwent labor induction as a prenatal examination revealed the woman was carrying a fetus with spina bifida. After a remarriage, the man and his current wife underwent labor induction as prenatal examination in the midtrimester revealed the woman was carrying a fetus with a meningocele. Both husband and wife had been in good health and neither of their extended families had a reproductive history of fetal malformations, genetic diseases, or exposure to adverse environmental factors. Laboratory tests were performed. G-banding of chromosomes showed no abnormalities in the husband or wife. In the husband, peripheral blood homocysteine level was 47.8 umol/L (reference value: 0–15 umol/ L) and folate level was 3.58 ng/mL (reference value: 3.1–19.9 ng/mL); evaluation of semen quality showed asthenospermia and sperm abnormalities; a sperm DNA fragment test showed a high proportion of sperm had significant DNA fragmentation; and analysis of the MTHFR gene revealed the MTHFR C677T homozygous TT genotype. In his current wife, peripheral blood homocysteine level was 8.10 umol/L (reference value: 0–15 umol/L) and folate level was 18.31 ng/mL (reference value: 3.1–19.9 ng/mL); and analysis of the MTHFR gene revealed the MTHFR C677T heterozygous CT genotype. The husband received homocysteine-lowering therapy. Three months later, his current wife became pregnant, and a healthy infant was spontaneously delivered at term.

### 2.2 Case 2

A 31-year-old man and his wife had 1 pregnancy affected by anencephaly and spina bifida that was discovered at 3 months of gestation, 1 biochemical pregnancy, and 1 embryonic pregnancy discovered at 7 weeks of gestation. Both husband and wife had been in good health and neither of their extended families had a reproductive history of fetal malformations, genetic diseases, or exposure to adverse environmental factors. Laboratory tests were performed. G-banding of chromosomes showed no abnormalities in the husband or wife. In the husband, peripheral blood homocysteine level was 64.2 umol/L and folate level was 4.7 ng/mL; evaluation of semen quality showed asthenospermia and sperm abnormalities; and analysis of the MTHFR gene revealed the MTHFR C677T homozygous TT genotype. In his wife, peripheral blood homocysteine level was 6.30 umol/L and folate level was 22.13ng/mL; and analysis of the MTHFR gene revealed the MTHFR C677T heterozygous CT genotype.

### 2.3 Case 3

A 31-year-old man and his wife underwent labor induction to terminate 3 pregnancies due to hydrocephalus, anencephaly, and cheilopalatognathus. Both husband and wife had been in good health and neither of their extended families had a reproductive history of fetal malformations, genetic diseases, or exposure to adverse environmental factors. Laboratory tests were performed. G-banding of chromosomes showed no abnormalities in the husband or wife. In the husband, peripheral blood homocysteine level was 54.0 umol/L and folate level was 3.96 ng/mL; and analysis of the MTHFR gene revealed the MTHFR C677T homozygous TT genotype. In his wife, peripheral blood homocysteine level was 7.90 umol/L and folate level was 16.71 ng/mL; and analysis of the MTHFR gene revealed the MTHFR C677T heterozygous CT genotype (Table 1 table 1).

**Table 1:**
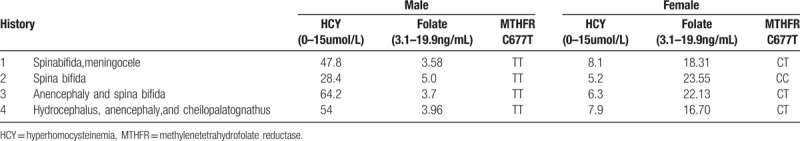
Summary of the case series.

## 3 Discussion

We present a case series of 3 males with a reproductive history of fetal NTDs. Cytogenetic examinations showed no chromosomal abnormalities in the husbands or their wives. A notable common characteristic was the presence of hyperhomocysteinemia combined with the MTHFR C677T homozygous TT genotype in the husbands. Although the pathogenesis of NTDs is complex, this case reports suggests an association between hereditary hyperhomocysteinemia in men and the occurrence of fetal NTDs. Importantly, both the former and current wife of 1 male with hyperhomocysteinemia combined with the MTHFR C677T homozygous TT genotype had a history of bearing a fetus with an NTD.

Evidence suggests that hyperhomocysteinemia in men affects sperm quality and DNA methylation. One study showed that folate level in seminal plasma is associated with sperm DNA damage, suggesting that low folate level in the sperm microenvironment may have an adverse effect on the stability of sperm DNA.^[14]^ Another study showed that an elevated homocysteine level in male semen can impair embryo quality in culture during in vitro fertilization/ intracytoplasmic sperm injection (ICSI).^[15]^ DNA methylation is an epigenetic modification that maintains chromosome structure, X chromosome inactivation, and gene imprinting,^[16]^ and human spermatogenesis is affected by changes in serum folate level.^[17]^ Low Vitamin B concentrations can cause elevated homocysteine concentrations and impaired remethylation of phospholipids, proteins, DNA, and RNA. These processes are essential for spermatogenesis, and disruption of this pathway may be detrimental to reproduction.^[14]^ Other reports concur that abnormal sperm DNA methylation increases the risk of fetal birth defects. Aitken and De Iuliis speculated that aberrant sperm DNA methylation may lead to epigenetic defects that adversely affect the first and subsequent generations of offspring.^[18]^

Findings from this case series and published literature imply that hereditary hyperhomocysteinemia in men affects sperm quality and sperm DNA methylation and causes epigenetic modifications that may adversely affect the fetus, resulting in NTDs. In this case series, 1 man with a reproductive history of fetal NTDs received homocysteine-lowering therapy, his wife became pregnant, and a healthy infant was spontaneously delivered at term. Although further research is required to substantiate our suppositions, we recommend monitoring homocysteine and folate levels in men before conception and supplementing with folate as needed, especially in men with a reproductive history of fetuses with neural tube or other birth defects.

## Author contributions

**Formal analysis:** Qingyang Shi, Yanhong Liu.

**Project administration:** Yanhong Liu.

**Resources:** Yueying Zhu.

**Validation:** Yang Yu.

**Writing – original draft:** Chunshu Jia.

**Writing – review & editing:** Yang Yu, Qingyang Shi, Yanhong Liu.
